# Intragroup Emotions: Physiological Linkage and Social Presence

**DOI:** 10.3389/fpsyg.2016.00105

**Published:** 2016-02-09

**Authors:** Simo Järvelä, Jari Kätsyri, Niklas Ravaja, Guillaume Chanel, Pentti Henttonen

**Affiliations:** ^1^Department of Information and Service Economy, School of Business, Aalto UniversityHelsinki, Finland; ^2^Department of Computer Science, School of Science, Aalto UniversityHelsinki, Finland; ^3^Department of Social Research and Helsinki Institute for Information Technology, University of HelsinkiHelsinki, Finland; ^4^Swiss Center for Affective Sciences, University of GenevaGeneva, Switzerland; ^5^Finnish Centre of Excellence in Intersubjectivity in Interaction, University of HelsinkiHelsinki, Finland

**Keywords:** psychophysiology, physiological linkage, emotions, social presence, emotional contagion

## Abstract

We investigated how technologically mediating two different components of emotion—communicative expression and physiological state—to group members affects physiological linkage and self-reported feelings in a small group during video viewing. In different conditions the availability of second screen text chat (communicative expression) and visualization of group level physiological heart rates and their dyadic linkage (physiology) was varied. Within this four person group two participants formed a physically co-located dyad and the other two were individually situated in two separate rooms. We found that text chat always increased heart rate synchrony but HR visualization only with non-co-located dyads. We also found that physiological linkage was strongly connected to self-reported social presence. The results encourage further exploration of the possibilities of sharing group member's physiological components of emotion by technological means to enhance mediated communication and strengthen social presence.

## Introduction

Emotional contagion—the tendency for emotions between two or more people to converge—is a well-established phenomenon (Barsade, 2002). Not only emotions displayed during face-to-face interaction but also mediated (text-based) emotional cues have been found to elicit similar emotions in others (Salminen et al., 2013). Physiological linkage—the synchronization of physiological activity across individuals—has been suggested as being one underlying mechanism of emotional contagion (Hatfield et al., 1993; Bruder et al., 2012). Bruder et al. (2012) suggested that in addition to physiological linkage, a social appraisal process exists, which also leads to emotional convergence within dyads. While the exact nature of emotions is still an open research question, it is widely agreed that emotions are complex constructs with several subcomponents: feelings, expressions and physiological states—and synchrony can occur on all these different levels. In the present study we investigated how technologically mediating two different components of emotion—expression and physiological state—to group members affects physiological linkage and various self-reports in a small group during video viewing. In different conditions the availability text chat (communicative expression) and visualization of group level physiological heart rates and their dyadic linkage (physiology) was varied.

### Physiological linkage

Psychophysiological measurements can provide real time data on the physiological states of participants that are directly related to their emotional states (Cacioppo et al., 2000) which in turn are affected by reactions to media (Ravaja, 2004). In a social group setting these methods can also be utilized in studying the effects of social dynamics on media experience. Physiological linkage refers to the extent to which physiological signals of two or more people are associated with each other, such as a mutual increase in heart rate (HR) during a shared experience. Physiological signals in general, but also linkage indices specifically, can provide information on the emotional and cognitive state of the user or a group of users that would normally remain unobservable.

The physiological linkage can occur through various processes. In addition to pure chance, the most common cause for joint changes in physiology within dyads in a given situation is simply the shared external stimulus to which they are both reacting in similar manner, e.g., the movie clip itself in a shared viewing situation. This type of physiological linkage is not the type of linkage we are currently interested in as it does not reveal anything about the connection of physiological linkage and social presence, or emotional contagion. Consequently this source of linkage should mostly be controlled in an experimental setup when studying other causes of linkage, as the causes cannot be distinguished from one another if they are all present. Bruder et al. (2012) distinguishes two other paths for emotional convergence that lead to physiological linkage: contagion-based and appraisal-based.

The mirror neuron system (Rizzolatti et al., 1996; Rizzolatti and Craighero, 2004) involved in imitating the perceived movements of other people, is a plausible neurophysiological mechanism underlying contagion-based physiological linkage. According to the embodied cognition theory (see Niedenthal et al., 2005; Barsalou, 2008; Mahon and Caramazza, 2008), in order to understand for example the facial expressions of others, the same brain areas of the observer must be activated that are used in producing them (Niedenthal et al., 2001). Mimicry of facial expressions as such is well-known phenomenon (e.g., Dimberg and Öhman, 1996; Korb et al., 2014), and within embodied cognition framework the basis of this phenomenon is that the perception of a smile causes similar brain activation as when the person would be smiling herself. This in essence means that the perception and mimicry of other person's emotional expressions leads to convergence of emotional states in the observer (Hatfield et al., 1993; Rizzolatti and Craighero, 2004). This in turn leads to the phenomenon of emotional contagion where emotional states are transferred from one person to another (Hatfield et al., 1993; Barsade, 2002; Bruder et al., 2012).

Appraisal-based emotional convergence occurs through social appraisal processes (Bruder et al., 2012) where individuals assess other people's emotional expressions in a given situation and based on them cognitively form a more shared understanding of the emotional situation, that then leads to emotional convergence, and the linkage of the physiological component of emotion. Appraisal-based path to emotional convergence assumably is less strongly connected to physiological linkage than contagion-based convergence, as it is not directly caused by physiological mimicry but is a result of higher social appraisal functions.

According to Emotions as Social Information (EASI) model, the social role of emotions is emphasized in ambiguous situations where the amount of more explicit social information is limited (Van Kleef, 2009, 2010; Van Kleef et al., 2010). Indeed, the amount of utilizable information is technologically mediated interaction is typically limited, and previous studies have found it to hinder the perception and mimicry of the other person's emotional states (Garcia et al., 1999). Often mediated interaction and communication takes place when participants are in physically separate locations, which naturally limits the channels of information, and the impact of merely being physically present with someone in the same space is cut off. Studies suggest however that even when physically separated, the social context and motives have an impact on emotional expressions and feelings (Hess et al., 1995; Jakobs et al., 1999a,b; Bruder et al., 2012). In such situations appraisal-based paths of convergence are arguably emphasized over contagion-based. Therefore, providing additional social information, such as expressive communicative social signals (e.g., text chat) and information on emotions through signal visualizations of physiological states (e.g., heart rates), may provide usable social information to an ambiguous situation and facilitate mimicry and emotional contagion between the participants.

Physiological linkage was first used in analysis of marital interactions, where several linkage indices were associated with conflict conversations (Levenson and Gottman, 1983). Later studies have shown that physiological linkage is also related to e.g., empathy (Levenson and Ruef, 1992, 1997) and performance (Henning et al., 2001). These studies highlight the fact that linkage is not associated only with negative interactions. A more recent suggestion is that linkage captures the intensity of social interactions that is elevated in, but not specific to, interpersonal conflicts (Chanel et al., 2012). Similarly, sense of social presence (Biocca and Harms, 2002)—a sense of dyadic interconnectedness or being together with other people in a given context—is linked to physiological linkage (Ekman et al., 2012; Järvelä et al., 2013).

### Present study

In the present investigation, we studied how providing socially utilizable information on two different components of emotion (expression and physiology) affected the emotional and social experiences of the participants, e.g., social presence, and especially physiological linkage between dyads during movie watching in small groups. Specifically, two different types of socially utilizable information and methods of mediating and presenting it through technological means were chosen for the experiment: (1) text chat on a second screen provided an expressive social information channel which was shared to all participants and only displayed information they contributed voluntarily, and (2) heart rate visualization displayed socially utilizable information on physiological state of the participants and their dyadic linkage continuously to the participants. Ordinarily physiological linkage between persons is not directly observable, but here a visualization of synchrony index that was measured continuously in real time was shown to the participants (see Methods for details). In a sense this enables social appraisal of a complex physiological state and appraisal-based emotional convergence. Such visualization enables the examination of whether the conscious acknowledgment of linkage has an effect on feelings of social presence and could increase emotional contagion within the group.

It has been found that although engaging in text chat during movie watching requires additional attention, it also increases liking and feeling of closeness within the group (Weisz et al., 2007). One of the key characteristics of text chat is asynchronous production where the actual writing of the message is not in synchrony with the actual interactive discussion; that is, the message is written first, sent later, and possibly read and replied by others sometime afterwards. This combined with the possibility for each contributor to write messages at the same time often splits chats into threads where replies to messages do not instantly follow but older messages can be replied to O'Neill and Martin (2003). This asynchronous nature of text chat makes it an activity that does not cause physiological linkage in group members just by providing rhythmic synchronous activity or stimulus—if chat increases physiological linkage it is because of its social aspects and the information it is used to communicate, that is, it is social appraisal-based.

Heart rate is one of the most common and arguably one of the most well-known measures in psychophysiology. Depending on the context, heart rate changes have been used to index increased attention, emotional arousal, and cognitive effort (Ravaja, 2004; Cacioppo et al., 2007). The synchronization of heart beats between two persons is a phenomenon which has been studied for example between patients and therapists, and between mothers and children (see Levenson and Ruef, 1997 for a review). Unlike most other neurophysiological measures, such as electroencephalography EEG or electrodermal activity etc., heart rate measures (e.g., beats per minute, BPM) are rather intuitive to understand and people commonly have a preliminary grasp on what they imply (e.g., arousal) (Janssen et al., 2013). This intuitiveness is essential when visualizing biosignals in an attempt to provide meaningful information to participants concerning their own physiological state. Intuitiveness allows using rather straightforward visualizations (e.g., beating heart icons for heart rate) whereas less intuitive ones would require more elaborated metaphorical visualizations (e.g., clock's hands moving at different speed representing different bands of EEG activity). Although heart rate visualization itself is probably not necessarily enough to cause heart beat synchronization by itself, it can mediate heart rate information to others and increase physiological linkage between two or more people through higher-order social processes. One possible channel for how biosignal visualizations would support physiological linkage is by increasing interoceptive awareness (Craig, 2002)—the awareness of your own bodily state—of all participants, and bringing their attention to the group process of linkage. It can be seen either as making contagion-based convergence easier by boosting interoceptive awareness, or by transforming physiological information into a form that can be utilized in social appraisals. Increased interoceptive awareness has also been found to amplify the experience itself (Dunn et al., 2010).

It would be expected that text chat evokes a sense of human connection and provides emotional cues and information regarding other people (e.g., their feelings toward the movie, opinions, and interests), thereby potentially leading to an increased similarity between the emotional states of the participants. This increased interconnectedness is assumed to manifest as higher reported social presence and physiological linkage. In accordance with previous studies (e.g., Wagner et al., 2015) we expect participants to report increased pleasantness and arousal when chatting as the possibility share emotions and to interact with group members is presumed to be positive experience by default, but HR visualization is not expected to produce such an effect by itself. However, heart rate visualization is expected to increase both physiological linkage and social presence as it provides socially utilizable information on emotional states of the participants. In accordance with the EASI model of emotion (Van Kleef, 2009, 2010; Van Kleef et al., 2010) which states that the social role of emotions is emphasized in ambiguous situations, a pronounced effect is expected in regard to both chat and heart rate visualization when the participants are not physically co-located and where the amount of other socially utilizable information is lower. When spatially separated, some information channels are not in use and the whole situation is more ambiguous and this heightens the importance of the social and emotional cues provided by either chat or heart rate visualization.

The results of this study provide insight on how intragroup emotions are influenced by sharing different emotion components between group members by technological means. It also explores if a visualization of a physiological signal is sufficient emotional information to increase dyadic physiological linkage. In addition, on a more applied level the results contribute to how shared media experience can be enhanced by providing features that strengthen the social presence between group members during media enjoyment. Media is increasingly often enjoyed in situations where the group members are physically in separate locations and they are interacting through technical means. As the second screen phenomenon where e.g., television viewers use tablets to enhance their media experience through various means (Courtois and D'heer, 2012), is quickly spreading, the technical solutions through which these features can be implemented are opening up.

## Methods

### Participants

Participants were 62 (21 males and 41 females) Finnish university students whose age ranged from 19 to 35 years (*M* = 24.2 years). All participants provided informed consent prior to the beginning of the experiment. Due to some participants not arriving at the experiment and data lost due to technical reasons, the number of subjects in different analyses varied from 52 to 57.

### Stimuli

The stimuli shown in the present study consisted of four video clips, whose duration ranged from 5 min 59 s to 6 min 16 s. The video clips contained no spoken narrative and they were selected to elicit varying emotional valence (unpleasant to pleasant) and arousal (calmness to excitement) levels. Themes consisted of religion (pleasant low-arousal), parkour (pleasant high-arousal), poverty (unpleasant low-arousal), and climbing (unpleasant high-arousal). Religion and poverty clips were selected from the movie “Baraka” (Magidson Films 1992; directed by Ron Fricke), and the parkour and climbing clips were obtained from YouTube Internet service (http://www.youtube.com).

### Procedure

**Figure 1:**
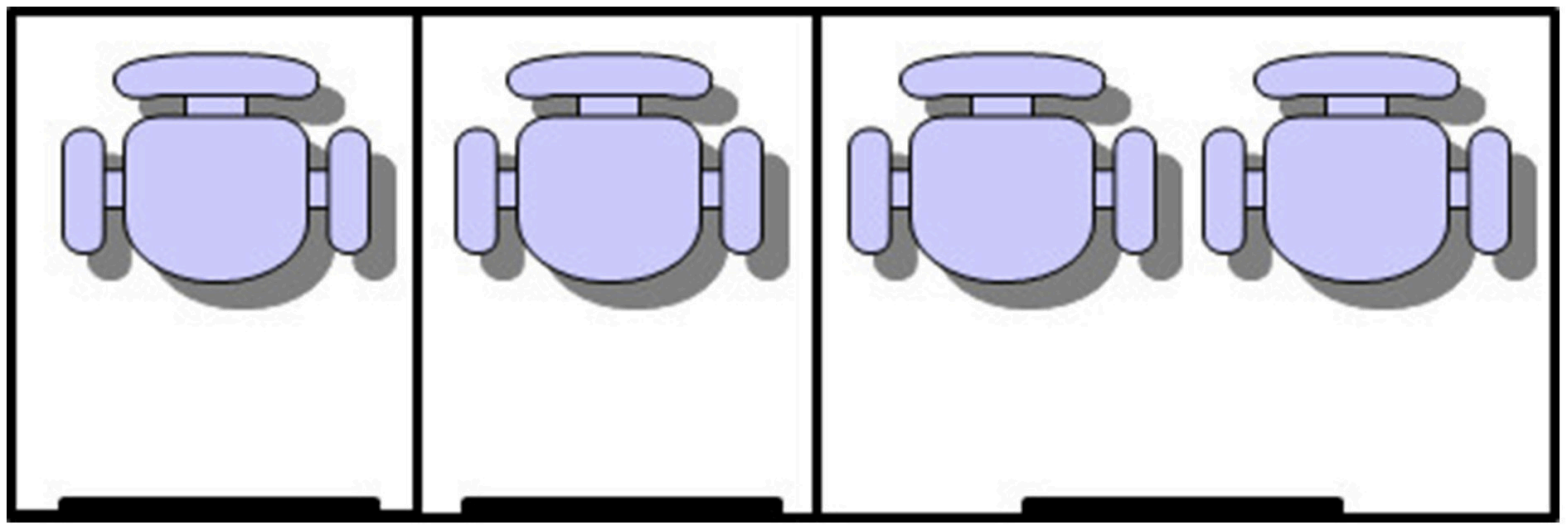
**The experimental setup**.

Participants arrived to the experiment in 16 four-participant groups. Two of the four participants formed one dyad at the beginning of the experiment so that one dyad was physically located in the same room, and the other two participants were located in two separate rooms. This setup of one co-located dyad and two separated individual participants aimed at comparing the effects of physical (co-located) and mediated (non-co-located) presence while they all were interacting together as a group (Figure 1).

Due to participant cancellations, three of the groups had only three participants. In one of these groups two participants were assigned to the same room (co-located condition), and in two groups two participants were assigned to different rooms (non-co-located condition). The remaining third subject took part in the experiment but was not included in the analysis. After instructions and a demonstration of the stimulus presentation system, baseline physiological measurements were recorded during a 5-min rest period. The participants were sitting in a chair facing the television screen (co-located dyad were sitting side by side) where video stimuli was presented in each room, and mobile devices were used for providing textual feedback from the participants. Participants listened to the video clip soundtracks via headphones, so that the co-located participants were not able to hear each other. The co-located dyad could see each other, but were mainly facing the television screen, and the individual participants in separate rooms did not have visual contact to other participants during the experiment.

**Figure 2:**
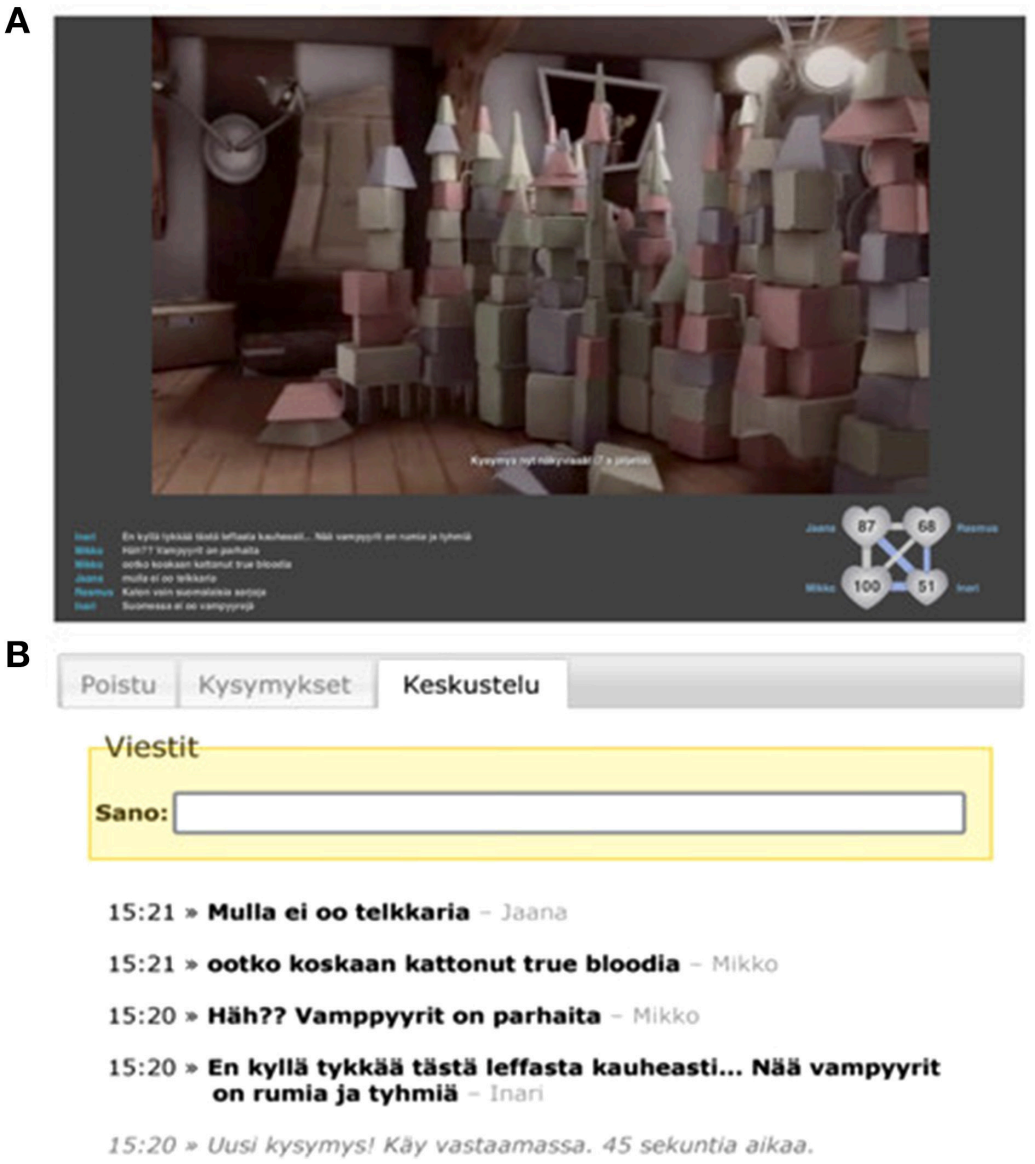
**The presentation view on the large screen when both chat and heart rate visualization are on (A) and the chat display on the mobile device (B)**.

For each group, the four video stimuli were assigned randomly to four display conditions defined by the inclusion of chat and heart rate (HR) displays (both off, only chat display on, only HR display on, and both on). The presentation order of conditions was randomized for all groups. During chat display, all the participants were able to read and write messages online (Figure 2B). During HR display, the heart rates of all participants, as well as the linkage between participant pairs, were displayed (Figure 2A). Linkage was defined as the correlation between a pair's HR signals, calculated on-the-fly within a 30-s moving time window. A 30-s time window was adopted in order to continuously visualize relatively recent changes in HR synchrony while allowing enough data points (typically, 20–60 heart beats) for calculating the correlation. The extent of HR correlation between each pair of participants was visualized with line thickness and color coding (gray for positive and blue for negative correlation; see Figure 2A).

To facilitate social interaction within the participant groups during stimulus presentation, questions related to the contents of each presented video clip (e.g., “Why do many religions encourage women to wear veils?”) were presented every 2 min (a total of three questions per video clip), participants were given a restricted amount of time (1 min 30 s) for providing their answers, and all answers were then displayed on the large screen until the beginning of next question (i.e., for 30 s). The facilitation was implemented to ensure at least a minimum amount of social interaction between the participants on a group level as the Finnish culture is not particularly extroverted.

After each video clip session, the participants filled a series of self-report questionnaires (see section Self-reports). After the questionnaires were filled by all participants, the next video was shown.

### Heart rate measurement and technical setup

The heart rates of all participants were measured with prototypical Polar Band heart rate monitors (Polar Electro; http://www.polar.fi). Videos were displayed on 40″ plasma TV screens, approximately 150–200 cm in front of the participants. Nokia N900 mobile devices (Nokia Corporation; http://www.nokia.com) operating on Maemo software platform (http://www.maemo.org) were used for presenting questions related to the video clips, providing answers to these questions, as well as for writing textual messages to other participants during chat display (see Section Procedure).

Stimulus presentation, HR and chat displays, and data collection were controlled with a specialized presentation system PRESEMO (see Figure 2; Kuikkaniemi et al., 2010), which has been developed for presenting various multimedia material (e.g., videos, text, and graphs) while allowing the audience to interact with the presentation and with each other via mobile devices. All data from Nokia N900 mobile devices and Polar Band monitors were transferred wirelessly over a Bluetooth connection to a centralized PRESEMO server (for further technical details, see ibid).

### Self-reports

#### Emotional evaluations

Participants rated their own emotional reactions to the video viewing sessions in terms of valence, arousal, and dominance on 9-step graphical scales. These scales were similar to Lang's (1980) Self-Assessment Manikin (SAM).

#### Interpersonal evaluations

The participants were asked to evaluate a series of 17 items measuring social presence (Biocca and Harms, unpublished)—that is, the degree to which they felt they were sharing a common experience—with their assigned (co-located or non-co-located) pair during video viewing. The following facets of social presence were measured: co-presence (e.g., “I often felt as if my partner and I were together in the same room”), attentional engagement (e.g., “I paid close attention to my partner”), emotional contagion (e.g., “I was sometimes influenced by my partner's moods”), comprehension (e.g., “I was able to communicate my intentions clearly to my partner”), and behavioral interdependence (e.g., “My actions were often dependent on my partner's actions”). For each 17 items they evaluated on 7 point scale (1 = I strongly disagree, 7 = I strongly agree). Participants also answered a series of eight questions measuring physical presence (e.g., “When the broadcast ended, I felt like I came back to the “real world” after a journey”) (Kim and Biocca, 1997). In contrast to social presence which refers explicitly to socially shared experiences, physical presence refers to the feeling of being physically present in the depicted virtual environment (Lee, 2004). To evaluate the sense of attraction with their pairs, participants were additionally asked to answer 13 questions on a 5-point scale, e.g., boring vs. interesting, cold vs. warm (Moreland and Beach, 1992). All of these scales have been shown to exhibit sufficient reliability (Moreland and Beach, 1992; Kim and Biocca, 1997; Harms and Biocca, 2004).

### Data pre-processing and analysis

A fundamental difference between the experimental HR visualization and the post-experimental HR data analysis was that the former was updated continuously on-the-fly, whereas the latter was done retrospectively for all the recorded data. HR measurements obtained from Polar Band devices were pre-processed in Matlab (version 7.10.0). HR data was first resampled to 32 Hz. Unrealistic values (3 standard deviations from mean, considering only values between 45 and 145 bpm) were replaced by interpolation. Cubic splines were used for both interpolation and resampling. Frequencies below 0.04 Hz were filtered out by removing a moving average cubic polynomial component from each individual data series. Resultant data series were smoothed with cubic polynomial in a 500 ms time window, and series mean was removed from each participant's data. When quantifying the HR linkage between the two members of each dyad, to allow some temporal lag between the physiological reactions, a ± 5-s temporal window was used in calculating the cross correlations (between the dyad members) for each HR sample (we assumed that the co-occurrence of physiological reactions with a longer temporal lag than 5 s is unlikely to be related to social processes). The highest cross-correlation value within this window was selected for the analysis. Mean cross-correlation coefficients were calculated separately for each film. Finally, Fisher transformation was applied to normalize the distribution of resultant values.

Conventional statistical methods such as analysis of variance (ANOVA) would not have been appropriate for the present data, which were hierarchical such that participants were nested within participant dyads, which were further nested within groups of two dyads. Instead, we adopted a multilevel modeling procedure that is a generalization of the more restricted ANOVA method (Quené and van den Bergh, 2004; Hoffman and Rovine, 2007; see (Hayes, 2006) for an excellent introduction on multilevel models), and which is particularly useful for the analysis of dyadic data (Kenny et al., 2006). Specifically, the data were analyzed by the Linear Mixed Models procedure in SPSS (version 18) with maximum likelihood estimation. With HR data, cross-correlations had been calculated for movie conditions that were presented repeatedly to subject pairs. Respectively, subject pair identifiers were specified as the subject variable and movie (four different movies) as the repeated variable. Unstructured variance-covariance structure (UN) was selected for the residuals based on best fit to the data (estimated with -2 log likelihood function). To account for the hierarchy of pairs within groups, a random intercept was specified with groups as the subject variable. A fixed-effects model was specified with main effects for movie (four levels), location (two levels: co-located and non-co-located), chat display (two levels: on, off), and HR display (two levels: on, off); as well as two 2-way interactions “location × chat display” and “location × HR display.”

Self-reports were available from all participants. Therefore, when analysing questionnaire data, participant identifiers were specified as the subject variable, and an additional random intercept was defined for subject pairs to account for the hierarchy between participants and pairs. The analysis remained otherwise identical to that of the HR data. When examining the association of self-reported social presence with HR cross-correlations, social presence scores were first averaged over both members in each pair and grand-mean centered. The HR data analysis was then repeated with a fixed-effects model that included only a main effect for this continuous covariate.

## Results

Results from LMM analyses for emotional and interpersonal evaluations are shown in Tables 1, 2, respectively.

**Table 1:** **Linear mixed model analyses for emotional evaluations**.

**Variable**	***df***	***F***	***p***
**VALENCE**			
Chat display	1, 151.46	27.26	< 0.001^***^
HR display	1, 149.06	0.03	0.864
Location	1, 13.24	0.23	0.639
Location × Chat	1, 148.88	0.83	0.365
Location × HR	1, 144.10	0.96	0.328
Video	3, 54.80	28.15	< 0.001^***^
**AROUSAL**			
Chat display	1, 146.21	10.53	0.001^**^
HR display	1, 118.18	0.16	0.687
Location	1, 27.07	0.66	0.423
Location × Chat	1, 145.35	0.03	0.854
Location × HR	1, 117.76	2.29	0.133
Video	3, 54.58	3.78	0.015^*^
**DOMINANCE**			
Chat display	1, 156.24	19.30	< 0.001^***^
HR display	1, 139.26	1.55	0.215
Location	1, 13.33	0.02	0.899
Location × Chat	1, 155.83	0.81	0.369
Location × HR	1, 138.19	0.24	0.625
Video	3, 54.83	0.69	0.565

* *p < 0.05*;

** *p < 0.01*;

*** *p < 0.001*.

**Table 2:** **Linear mixed model analyses for interpersonal evaluations**.

**Variable**	***df***	***F***	***p***
**ATTRACTION**			
Chat display	1, 151.39	44.62	< 0.001^***^
HR display	1, 123.36	1.87	0.174
Location	1, 27.60	2.48	0.127
Location × Chat	1, 150.15	2.23	0.137
Location × HR	1, 122.84	0.07	0.790
Video	3, 55.85	4.58	0.006^**^
**PHYSICAL PRESENCE**			
Chat display	1, 151.84	14.76	< 0.001^***^
HR display	1, 152.97	1.213	0.272
Location	1, 56.93	0.00	0.969
Location × Chat	1, 152.45	4.59	0.034^*^
Location × HR	1, 152.63	1.24	0.268
Video	3, 52.81	3.34	0.026^*^
**CO-PRESENCE**			
Chat display	1, 160.06	63.51	< 0.001^***^
HR display	1, 133.51	0.02	0.895
Location	1, 45.49	5.36	0.025^*^
Location × Chat	1, 158.27	27.38	< 0.001^***^
Location × HR	1, 130.28	1.01	0.316
Video	3, 54.28	0.49	0.688
**PERCEIVED ATTENTIONAL ENGAGEMENT**			
Chat display	1, 150.87	21.04	< 0.001^***^
HR display	1, 148.08	0.08	0.775
Location	1, 9.88	0.92	0.360
Location × Chat	1, 151.47	2.01	0.158
Location × HR	1, 147.64	1.45	0.231
Video	3, 55.95	0.70	0.558
**PERCEIVED EMOTIONAL CONTAGION**			
Chat display	1, 150.18	72.43	< 0.001^***^
HR display	1, 122.82	1.34	0.249
Location	1, 57.10	6.98	0.011^*^
Location × Chat	1, 149.49	3.26	0.073
Location × HR	1, 122.33	0.28	0.595
Video	3, 55.56	0.86	0.469
**PERCEIVED COMPREHENSION**			
Chat display	1, 144.52	549.55	< 0.001^***^
HR display	1, 119.99	0.06	0.809
Location	1, 25.12	10.95	0.003^**^
Location × Chat	1, 144.55	0.99	0.322
Location × HR	1, 119.47	0.23	0.633
Video	3, 54.75	2.11	0.110
**PERCEIVED BEHAVIORAL INTERDEPENDENCE**			
Chat display	1, 154.29	181.93	< 0.001^***^
HR display	1, 145.31	0.05	0.824
Location	1, 26.06	0.49	0.489
Location × Chat	1, 153.99	1.50	0.223
Location × HR	1, 144.41	0.09	0.769
Video	3, 56.24	4.01	0.012^*^

* *p < 0.05*;

** *p < 0.01*;

*** *p < 0.001*.

### Manipulation checks

Videos exerted significantly different effects on emotional valence and arousal ratings (Table 1). Pairwise *post-hoc* comparisons with Bonferroni correction confirmed that *a priori* pleasant videos (religion: *M* = 6.3; parkour: *M* = 6.2) elicited higher valence ratings than *a priori* unpleasant videos (poverty: *M* = 4.2; climbing: *M* = 5.5; *SE* = 0.2 for all videos). In contrast, *a priori* high-arousal videos (parkour: *M* = 4.6, *SE* = 0.2; climbing: *M* = 4.8, *SE* = 0.3) failed to elicit significantly higher arousal ratings than *a priori* low-arousal videos (religion: *M* = 4.1, *SE* = 0.2; poverty: *M* = 5.1, *SE* = 0.2). To control for any confounds caused by the different videos, the main effect of video was retained in all analyses. Significant video effects emerged for some variables (Table 2). *Post-hoc* comparisons showed that the poverty video elicited higher attraction ratings than climbing and parkour videos (*M*s = 3.5, 3.3, and 3.3), higher physical presence than parkour video (*M*s = 2.8 and 2.4), and higher perceived behavioral interdependence ratings than parkour video (*M*s = 2.2 and 1.8, *SE* = 0.1 for all effects). Preliminary analyses showed that interactions between the video condition and chat display, HR display, and location conditions were non-significant for all dependent variables (*p*s > 0.05).

### Emotional evaluations

**Figure 3:**
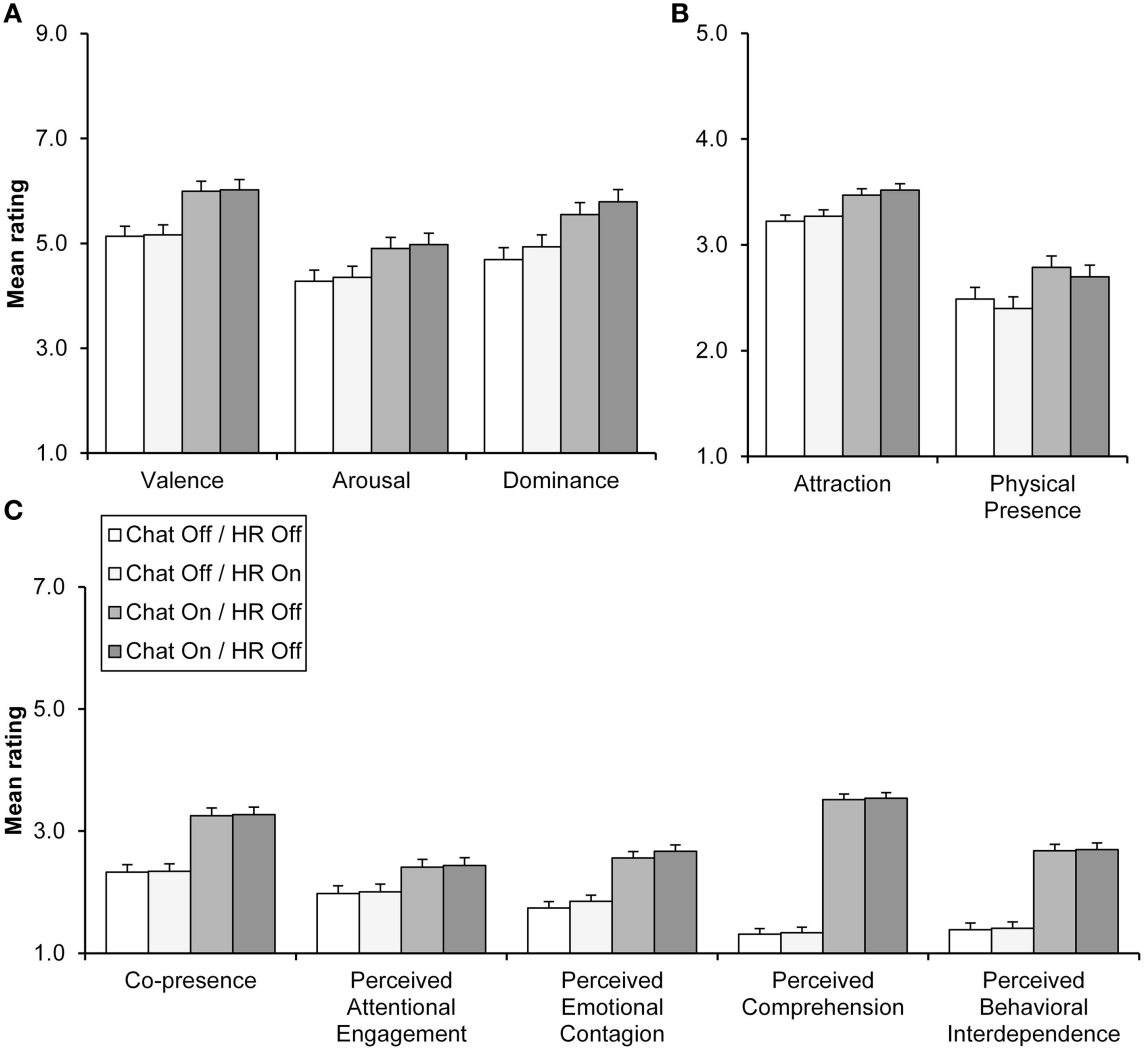
**Mean evaluations for emotional (A) and interpersonal evaluations (B,C) by chat and HR display conditions**. Interpersonal evaluations are shown separately for variables measured on 5-step **(B)** and 7-step scales **(C)**. Error bars refer to standard errors of the mean.

**Figure 4:**
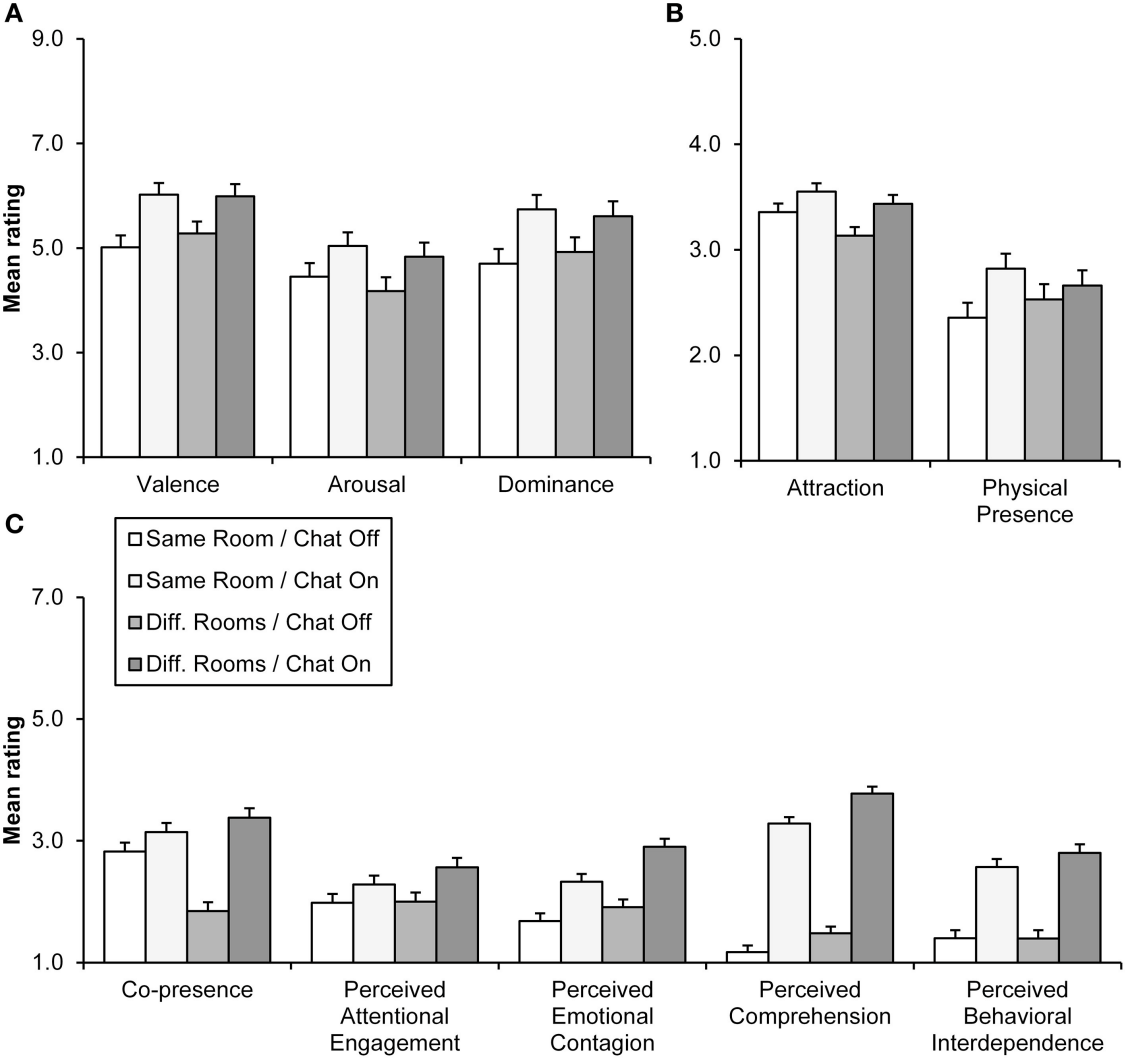
**Mean evaluations for emotional (A) and interpersonal evaluations (B,C) by location and chat display conditions**. Interpersonal evaluations are shown separately for variables measured on 5-step **(B)** and 7-step scales **(C)**. Error bars refer to standard errors of the mean.

Chat display exerted significant effects on all emotional evaluations (Table 1). Mean evaluations for emotional evaluations in chat and HR display conditions can be seen in Figure 3A. The participants reported feeling more aroused, in control of the situation, and more pleasant, when chat was available. In contrast, the HR visualization did not have a similar effect on these components. The location of pairs did not interact significantly with the chat or HR (see Figure 4A and Table 1) display conditions.

### Interpersonal evaluations

Social presence subscales all showed sufficient reliability (co-presence, Cronbach's α = 0.77, 4 items; Perceived Emotional Contagion, α = 0.90, 4 items; Perceived Comprehension, α = 0.92, 3 items; and Perceived behavioral interdependence, α = 0.89, 3 items) except for the 3 item Perceived Attentional Engagement subscale, that had Cronbach'sα = 0.57. Despite relatively low reliability, scores for Perceived Attentional Engagement are still reported here as they showed very similar results to other social presence subscales. Physical Presence (α = 0.87, 8 items) and Attraction (α = 0.87, 13 items) scales showed high reliability.

Chat display exerted significant effects for all interpersonal evaluations, whereas the effects of HR display were all non-significant (Table 2). As can be seen in Figures 3B,C, chat display elicited greater attraction, physical presence, and social presence (as measured by all of the five social presence subscales) ratings.

A significant interaction between location and chat display (Table 2) demonstrated that the effect of chat display on co-presence evaluation was more pronounced with the non-co-located pairs (Figure 4C). In contrast, there were no significant interactions between location and HR display (see Table 2). Contrary to expectations, chat display increased evaluated physical presence more with co-located rather than with non-co-located pairs (Table 2 and Figure 4B), but this is possibly because increased social presence also emphasized the physical presence of being in the same room. Unexpectedly, non-co-located pairs reported significantly greater social presence as measured with emotional contagion and comprehension subscales (Table 2 and Figure 4C). However, for emotional contagion there was also a non-significant trend (*p* < 0.10) toward a greater chat display effect for non-co-located pairs. Given that this effect was similar to that of co-presence, it is possible that these location main effects may have stemmed from interaction effects between location and chat display.

### Physiological linkage

**Figure 5:**
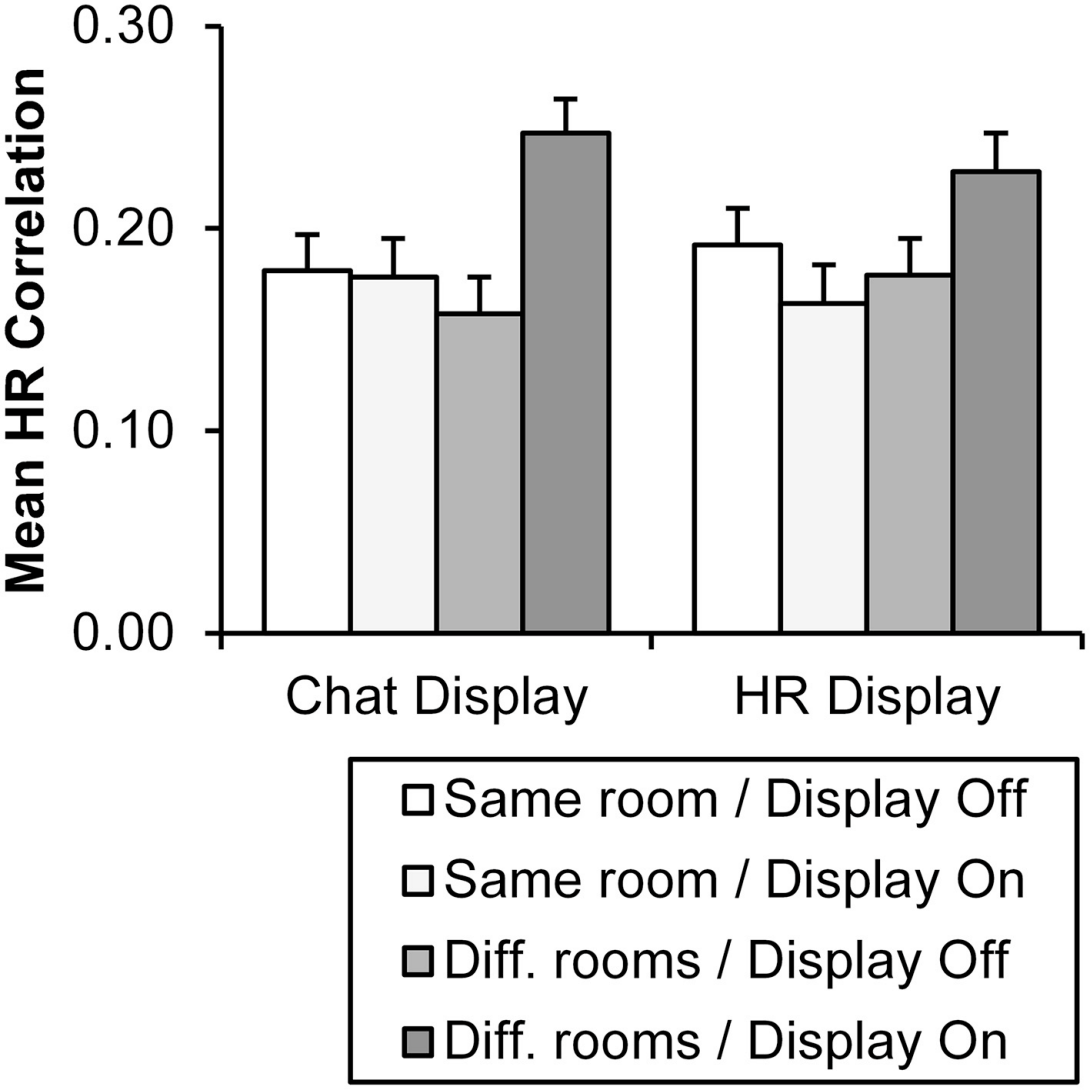
**Mean HR cross-correlation values by location and display conditions**. Display conditions are shown separately for chat and HR displays. Error bars refer to standard errors of the mean.

Mean HR cross-correlations between subject pairs for chat and HR display conditions are presented in Figure 5. The main effect of chat display, as well as the interaction effects between location and chat display and location and HR display were significant (Table 3). In general, HR cross-correlations were higher when the chat display was enabled but were not affected by the HR display. Importantly, however, both the effects of chat and HR displays were more pronounced for the non-co-located pairs (Figure 5). Simple effect analyses for non-co-located pairs indicated significant chat display, *F*_(1, 53.20)_ = 17.26, *p* < 0.001, and HR display effects, *F*_(1, 58.41)_ = 4.32, *p* = 0.04. With co-located pairs, non-significant effects were observed for both chat, *F*_(1, 60.23)_, = 0.02, and HR displays, *F*_(1, 62.22)_ = 1.41. Table 4 displays statistical tests for the associations between HR cross-correlations and the social presence evaluations. The results demonstrated that the HR synchrony between participant pairs showed a significant positive correlation with all evaluated social presence scales, which emphasizes how social presence and physiological linkage are connected.

**Table 3:** **Linear mixed model analyses for heart rate cross-correlations**.

**Variable**	***df***	***F***	***p***
HR display	1, 49.86	0.38	0.538
Chat display	1, 43.41	7.32	0.010^*^
Location	1, 5.77	2.45	0.170
Location × HR	1, 60.24	5.38	0.024^*^
Location × Chat	1, 60.29	8.54	0.005^**^
Video	3, 21.56	0.27	0.845

* *p < 0.05*;

** *p < 0.01*.

**Table 4:** **Linear mixed model analyses for the associations between HR cross-correlations and social presence evaluations**.

**Variable**	***df***	***F***	***p***	**Parameter estimate**	***SE***
Co-presence	92.04	5.13	0.026^*^	0.03	0.01
Attentional engagement	91.48	5.73	0.019^*^	0.04	0.02
Emotional contagion	83.63	4.73	0.032^*^	0.03	0.01
Comprehension	83.05	7.85	0.006^**^	0.02	0.01
Behavioral interdependence	86.26	5.30	0.024^*^	0.03	0.01

* *p < 0.05*;

** *p < 0.01*.

## Discussion

In our experiment we set out to examine how providing more socially utilizable emotional information to participants in a group media consumption situation would affect their experience. The text chat option provided sporadic voluntary communicative emotional expressions to the group while heart rate visualization showed continuous involuntary information on group member's physiological state and their dyadic linkage to other group members.

In this setting, text chat was clearly an effective method of affecting the experience. The subjects reported higher feelings of valence, arousal, attraction, social, and physical presence. The HR visualization by itself did not have such an effect. Simple biosignal visualization of group members' physiological state was not enough to significantly affect self-reported feelings. However, HR visualization and text chat both increased physiological linkage (heart rate cross-correlation) when the participants were physically non-co-located. The idea of physiological linkage as a measure of social presence was supported as they were positively correlated with every subscale of self-reported social presence. These results are in accordance with our initial hypotheses, except that HR visualization had weaker effects throughout than expected. When physically co-located, the effects were weaker, especially with HR visualization, which did not have noticeable effect. Our interpretation for this is that it was because the visualization of a physiological component of emotion is harder to interpret and of lesser information value than expressive social signals such as text chat. A possible interpretation for the lack of effect for HR visualization in co-located condition is that contagion-based emotional convergence is a more natural path to utilize the information on physiological state, but that path was already fully in operation when the participants were physically co-located, thus the visualization did not provide anything more by converting the physiological state into form that social appraisal processes can utilize. Another possibility is that in non-co-located condition when the amount and type of social information is lesser, those that are available are emphasized, and consequently the HR visualization is more effective when other forms of information are not available. This would also explain why HR synchrony was higher in non-co-located situations and not just close to co-located situation. It can be concluded that the availability of socially utilizable information, whether it was text chat or HR visualization, increased physiological linkage and associated social presence when the amount of socially utilizable regular information was low, e.g., when communication between subjects was only by technological means. This finding supports the EASI model of emotion, which states that the social role of emotions is emphasized in ambiguous situations. The solid connection between physiological linkage and self-reported social presence supports the idea that social presence could, at least partly, be the subjective feeling component of physiological linkage—however this hypothesis naturally requires further research into the topic.

There were some challenges during the process, mainly with the data quality of the consumer grade heart rate monitor, which is why the data had to be processed rather heavily before the analysis. With a higher quality data, more advanced HR indices could have been calculated and a shorter time window used for linkage calculations. In general, the quality of the non-filtered heart signals might explain the absence of effects of the HR visualization. However, the data quality was sufficient for the results presented here Optimally, the number or participants should have been larger to compensate for the small effect sizes. Now some of the results lack statistical power, and perhaps even more solid results would have been acquired with a larger sample size. Also, as the two information types (text chat and HR visualization) varied in more ways than one (e.g., voluntary vs. involuntary, real-time vs. delay, sporadic vs. continuous) exact interpretations for the results are difficult. Also, the HR visualization provided information not only on the heart rates of group members but also the dyadic linkage between them, and it is impossible to separate the effects from each other. In addition, in a sense linkage scores were used both as dependent and independent variables in the setup. We might not be able to precisely say what caused the difference between chat and HR visualization, but examining those two still provides us with an overview of how a typical mediated communication affects social and emotional states and also how it can be still enhanced with less common biosignal visualizations providing usable emotional and social information.

The positive results acquired in this experiment raise several questions for future studies. For example would a different type of heart rate visualization or a different biosignal altogether produce different effects? Or does the type of emotion (e.g., positive or negative valence, different discrete emotions) experienced affect how physiological synchrony is associated with feelings of social presence? How would the manipulation of the social context (e.g., cooperation vs. competition) affect physiological linkage? Can different paths of convergence be experimentally separated, e.g., do they require different time scales to operate?

Overall, our interpretation is that technological augmentation provides emotional cues and socially utilizable information, and affects intragroup emotions especially when regular communication is somehow limited. For example, text chat is effective when talking is prohibited or considered disturbing (like during movie watching), and sharing indices of group's shared physiological synchrony is effective when the group members are physically separated from each other. In a sense, these technical solutions compensate for the lack of emotional cues and information that exist in typical face-to-face communication. Their promising potential for augmenting various group situations should be further studied and experimented with. In addition to providing practical solutions for modern technologically mediated communication, this line of research will reveal more fundamental dynamics how group-level emotional expressions and their sharing affects group emotions, and how they manifest in physiological responses and their synchrony.

## Author contributions

GC designed the study and performed the experiment, PH pre-processed the data and calculated synchrony indices, JK analyzed the data and wrote the manuscript with SJ and NR.

### Conflict of interest statement

The authors declare that the research was conducted in the absence of any commercial or financial relationships that could be construed as a potential conflict of interest.
